# Supplementary material for: Unraveling surface and bulk dynamics of iron(III) molybdate during oxidative dehydrogenation using operando and transient spectroscopies
Source: Commun Chem. 2023 Oct 26;6:230. doi: 10.1038/s42004-023-01028-8 (PMC10603085; doi:10.1038/s42004-023-01028-8)
Supplement: Supplementary file 2 — Supplementary Material [file 42004_2023_1028_MOESM2_ESM.pdf]

Supporting Information

Unraveling Surface and Bulk Dynamics of Iron(III) Molybdate during Oxidative Dehydrogenation using Operando and Transient Spectroscopies

Leon Schumacher, Mariusz Radtke, Jan Welzenbach, Christian Hess*

Technical University of Darmstadt, Department of Chemistry, Eduard-Zintl-Institut für Anorganische und Physikalische Chemie, Peter-Grünberg-Str. 8, 64287 Darmstadt, Germany

*Corresponding Author (E-Mail: christian.hess@tu-darmstadt.de)

1. Additional Spectroscopic Data

Supplementary Table 1: Characterization data for $Fe_2(MoO_4)_3$, MoO_x/Fe_2O_3, and MoO_3.

Sample	ICP-OES Mo/Fe bulk ratio	N_2 Physisorption Surface Area	SEM Imaging Particle Size
$Fe_2(MoO_4)_3$	1.53	2.9 m²/g	2-8 µm
MoO_x/Fe_2O_3	0	8.7 m²/g	5 µm
MoO_3	0	3.9 m²/g	2-5 µm

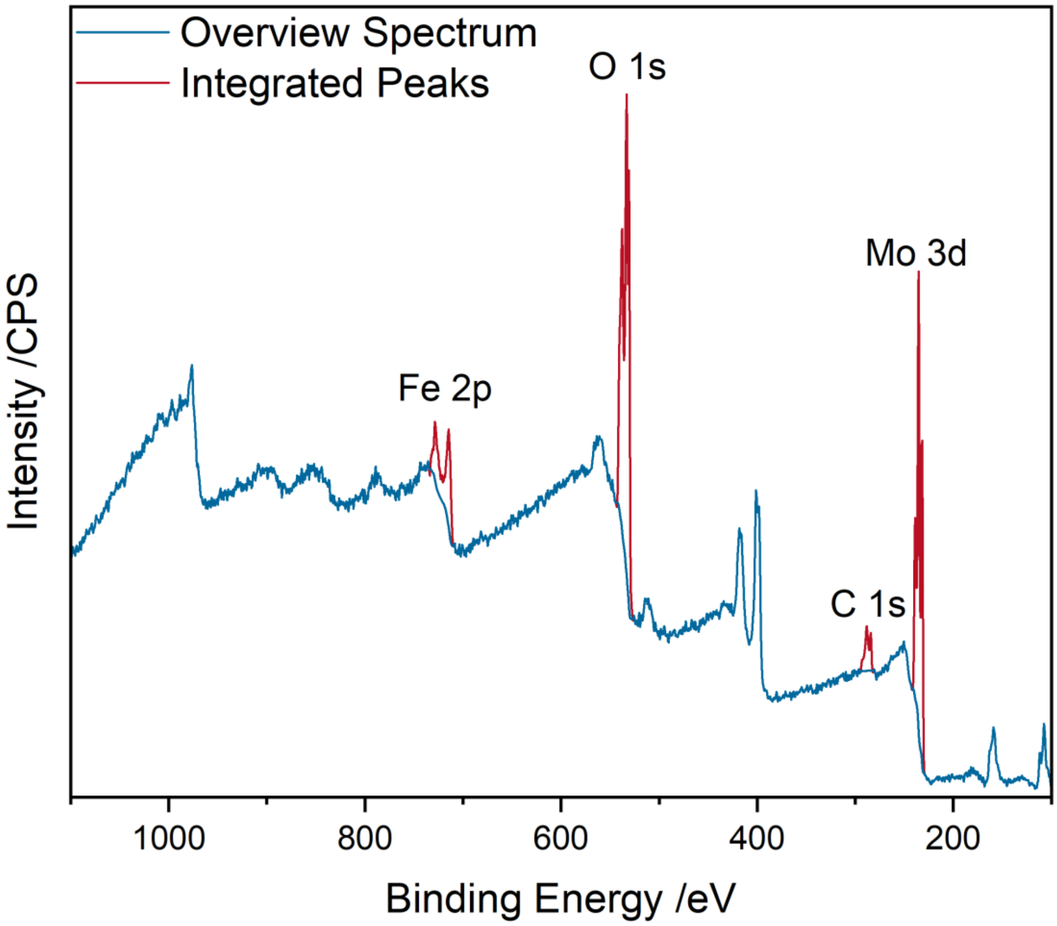

Supplementary Figure 1: Overview XP spectrum of $Fe_2(MoO_4)_3$. The peaks used for quantification (Supplementary Table 2) are highlighted in red.

Supplementary Table 2: Elemental composition of $Fe_2(MoO_4)_3$ based on X-ray photoelectron (XP) survey spectra and the use of the relative sensitivity factor (RSF) values from Table 1.

Fe	Mo	O	C	Mo/Fe
6.2	15.6	60.5	17.7	2.5

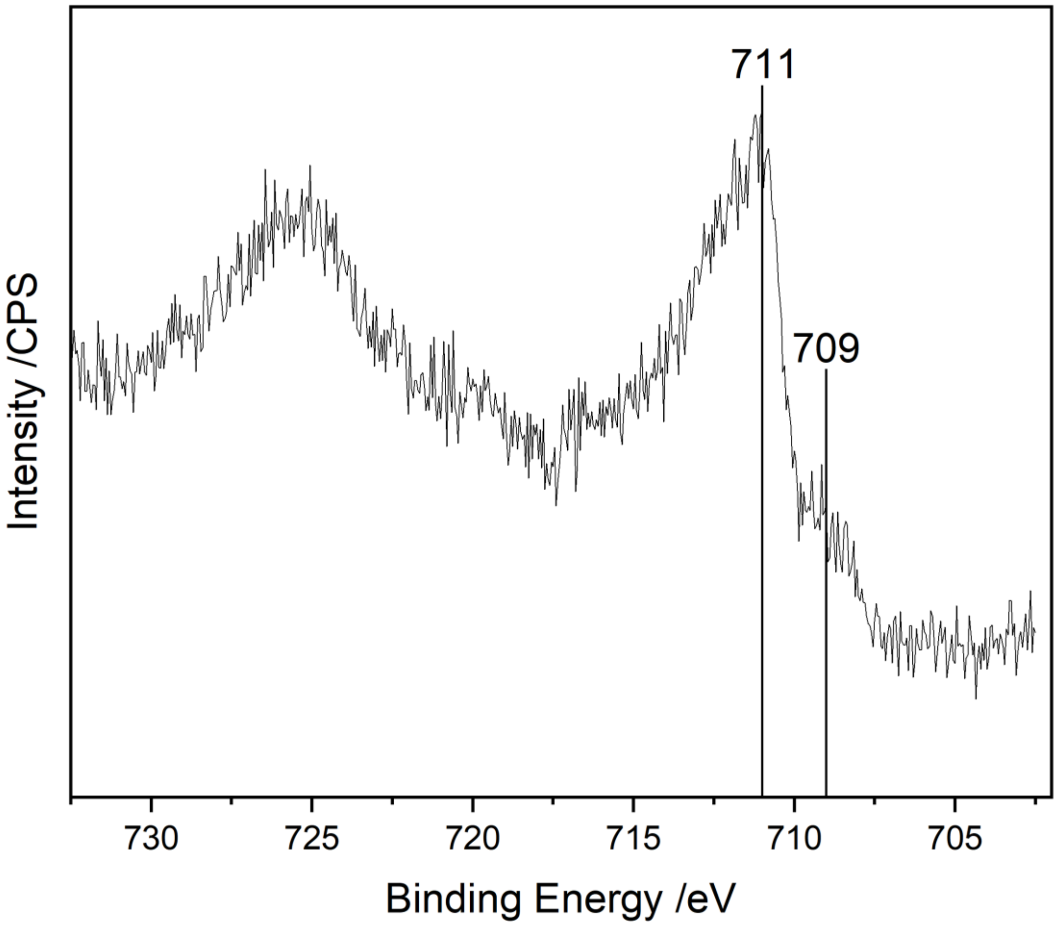

Supplementary Figure 2: Fe $2p_{3/2}$ photoemission of the $Fe_2(MoO_4)_3$ sample used in this study. The positions of characteristic Fe peaks are marked.

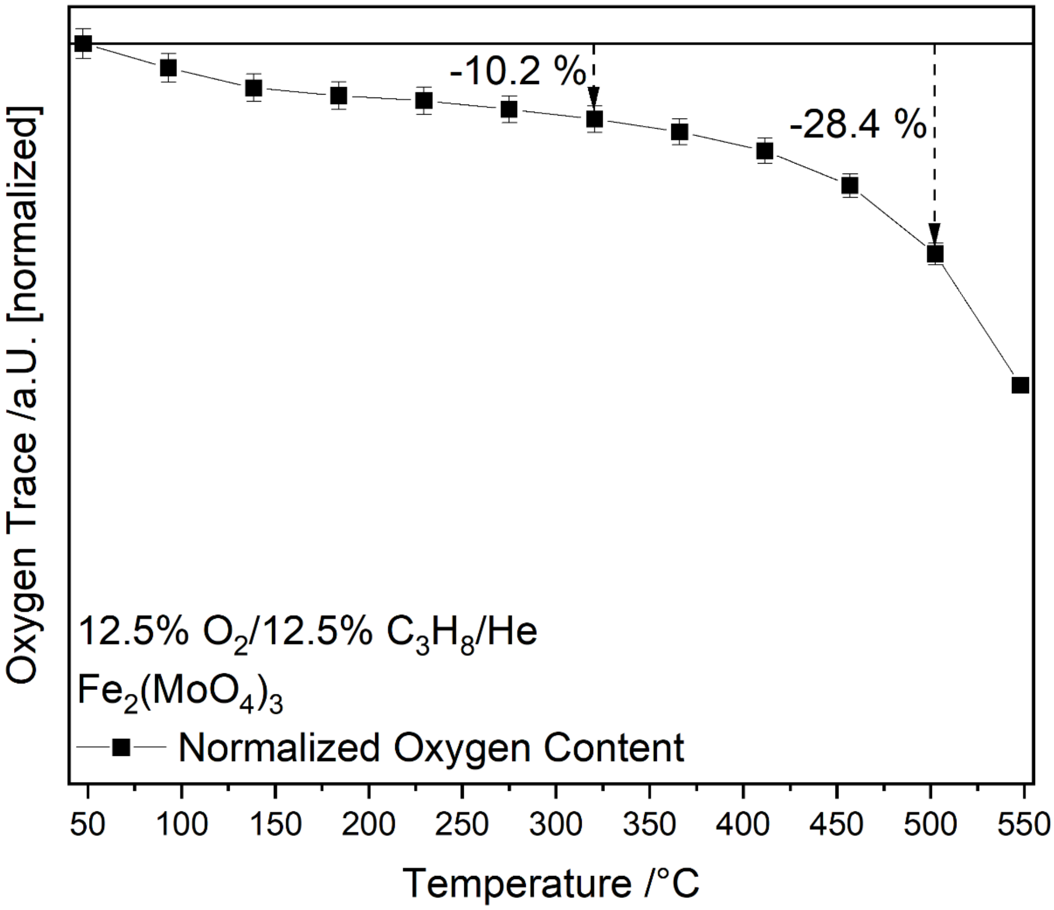

Supplementary Figure 3: Oxygen consumption of $Fe_2(MoO_4)_3$ during reaction conditions at different temperatures. The temperatures used for operando experiments are highlighted and the percentage of consumed oxygen in regard to the amount where no consumption occurs is indicated by dashed arrows. The error bars were produced as a percentage of the areas obtained from the GC by error propagation.

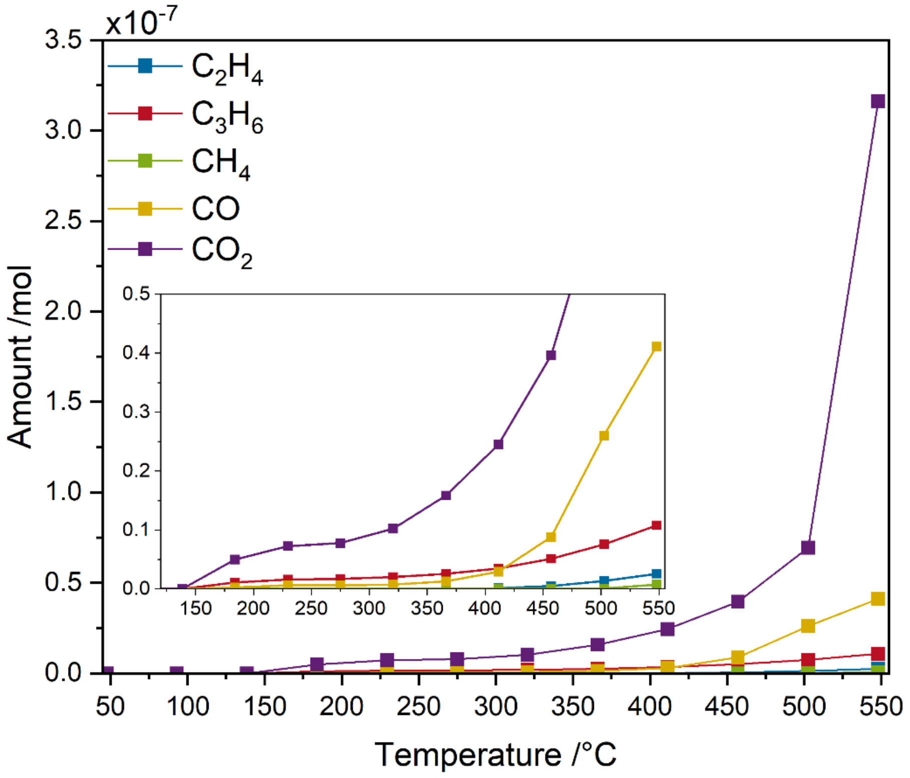

Supplementary Figure 4: Molar product distribution of all products detected by the gas chromatography (GC) during the treatment of $Fe_2(MoO_4)_3$ in 12.5% O_2/12.5% C_3H_8/He between 50 and 550 °C after initial dehydration in 12.5% O_2/He at 365 °C. The inset gives an enlarged view of the temperature region between 120 and 550 °C.

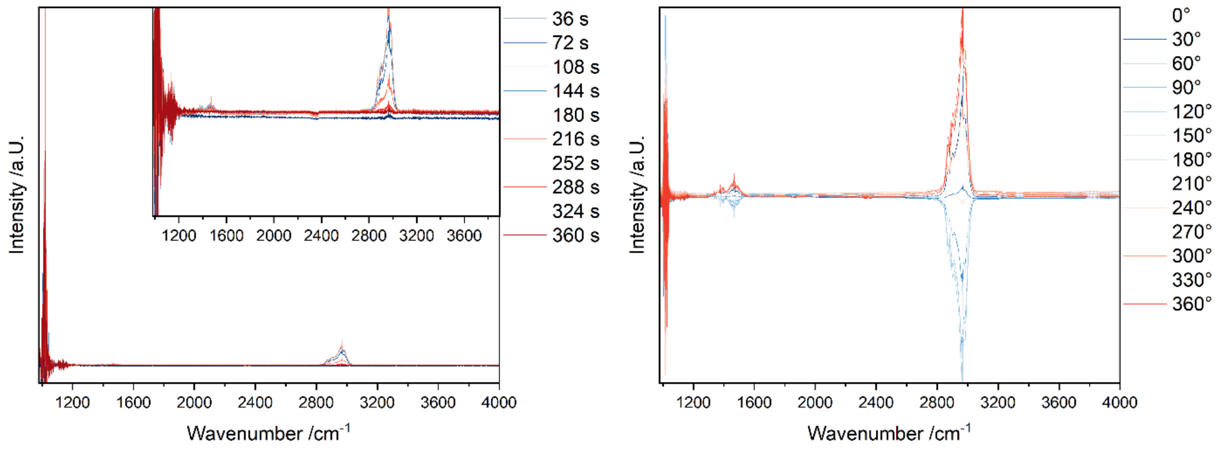

Supplementary Figure 5: Comparison between the time domain (before PSD, left) and phase domain (after PSD, right) DRIFT spectra of $Fe_2(MoO_4)_3$ recorded under modulation-excitation conditions as described in the experimental section.

Supplementary Discussion 1

The time resolved DRIFT spectra of $Fe_2(MoO_4)_3$ were recorded under a constant propane (12.5% C_3H_8/He) and a pulsed oxygen (12.5% O_2/12.5% C_3H_8/He) flow. A spectrum was measured every 1.54 s while the gas phases were switched according to the experimental section. Due to the reaction conditions being switched on and off, the signal of peaks that participate in the reaction follow the periodic pertubation with a certain phase-shift, while peaks that do not participate in the reaction have no periodic behavior. When the results are Fourier transformed over the entire phase space (phase sensitive detection, PSD), only those peaks following the periodic pertubation due to a concentration modulation remain, while the remaining signals are eliminated, allowing for the discrimination between active and observer species. Due to this, the detection sensitivity towards weak signals that are either heavily overlapped by spectator species/background noise or participate only weakly in the reaction, is significantly enhanced.[1,2]

This is evidenced by Supplementary Figure 5, where, in the time-resolved spectra (left), a large but noisy signal of the Mo=O region between 950 and 1080 cm^{-1} is observed, but no significant changes between the time resolved spectra can be detected, while a constant background overlaps some of the propane gas phase peaks between 1200 and 1600 cm^{-1}. After PSD, the propane gas phase is more pronounced, but a participation of the Mo=O peak is still observable, indicating its importance in the reaction. To emphasize the assignments to the propane gas phase and to ensure that,

besides the gas phase, only the Mo=O peak participates, the same measurement as in Figure 2 and Supplementary Figure 5 is performed in the same manner over KBr, which is IR transparent and shows no conversion. The results after the PSD are shown in Supplementary Figure 6.

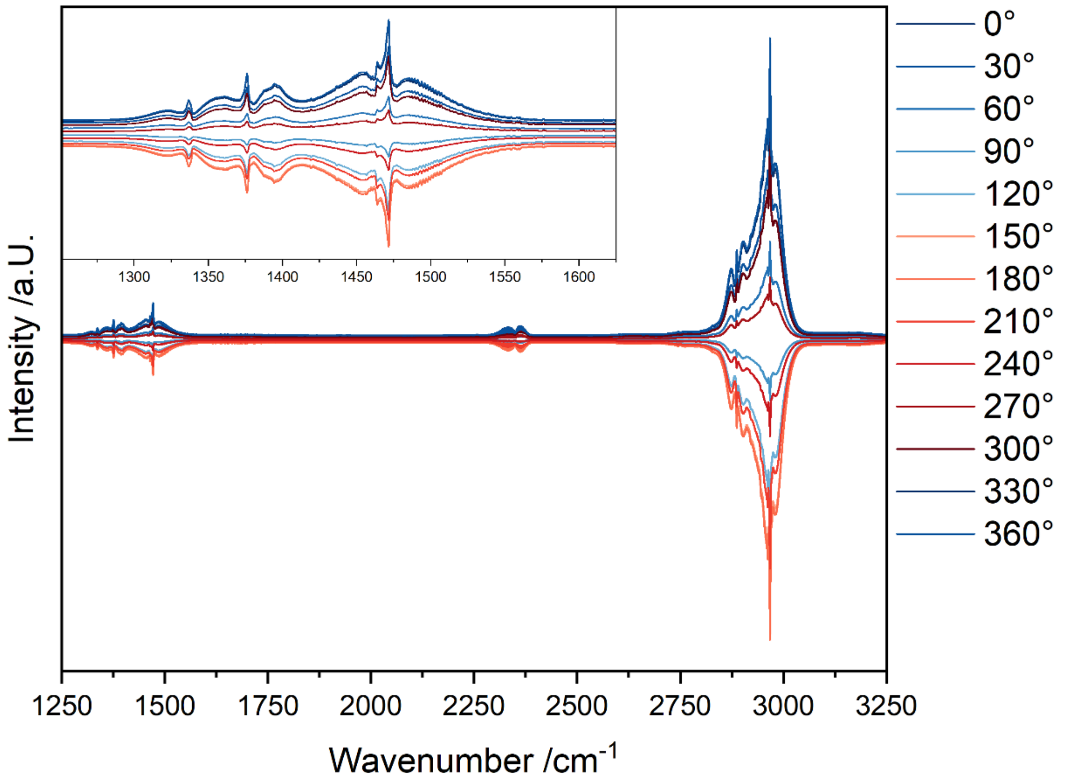

Supplementary Figure 6: Gas phas contribution from propane to the ME-DRIFT spectra afer PSD.

Comparing the results in Supplementary Figures 5 and 6, we can deduce that only propane gas phase contributions can be observed beside the Mo=O signal in the ME-DRIFT spectra of $Fe_2(MoO_4)_3$.

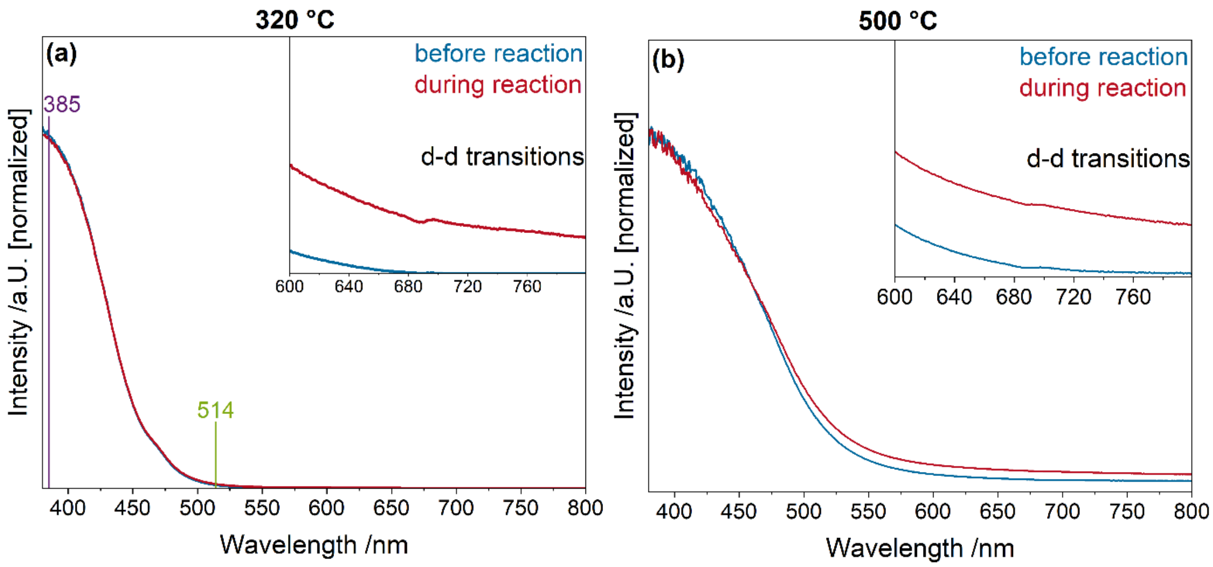

Supplementary Figure 7: Operando UV-Vis spectra of $Fe_2(MoO_4)_3$ recorded in a feed of 12.5% O_2/12.5% C_3H_8/He, compared to oxidative conditions (12.5% O_2/He) at **(a)** 320 and **(b)** 500 °C. Spectra have been normalized for comparability. The insets give an enlarged view of the absorption between 600 and 800 nm. The wavelengths at which Raman spectroscopy was performed are marked in the left panel.

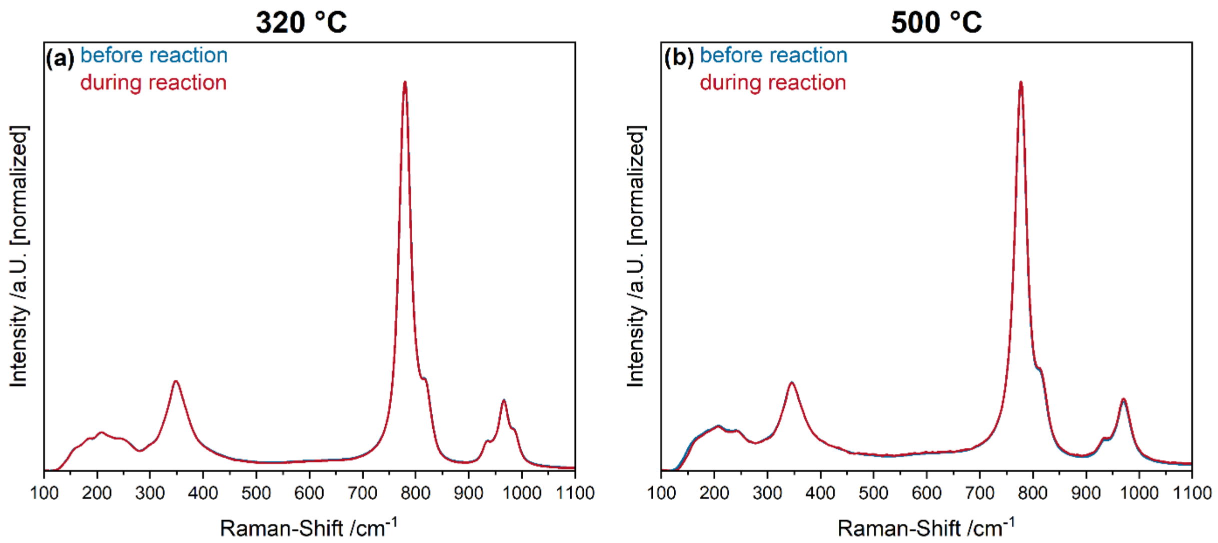

Supplementary Figure 8: Operando Raman spectra (at 514 nm excitation) of $Fe_2(MoO_4)_3$ recorded in a feed of 12.5% O_2/12.5% C_3H_8/He, compared to oxidative conditions (12.5% O_2/He) at **(a)** 320 °C and **(b)** 500 °C. Spectra have been normalized for comparability.

Supplementary Table 3: Assignment of the Raman features detected for $Fe_2(MoO_4)_3$.[3]

Wavenumber /cm^{-1}	Assignment
117 – 258	Lattice modes
299 – 370	MoO_4 bending modes
785 – 823	MoO_4 antisymmetric stretching modes
938 – 991	MoO_4 symmetric stretching modes

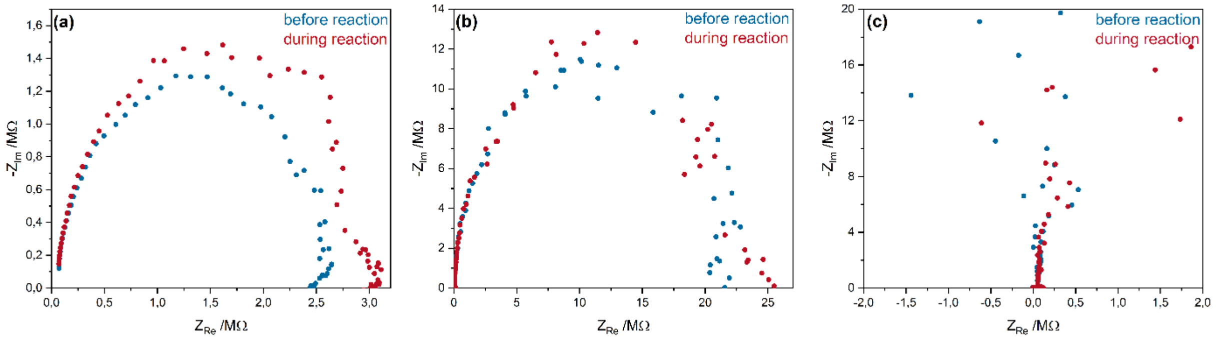

Supplementary Figure 9: Operando impedance spectra of **(a)** $Fe_2(MoO_4)_3$, **(b)** 1.0MoO_x+Fe_2O_3, and **(c)** MoO_3 at 320 °C in a feed of 12.5% O_2/12.5% C_3H_8/He, compared to oxidative conditions (12.5% O_2/He).

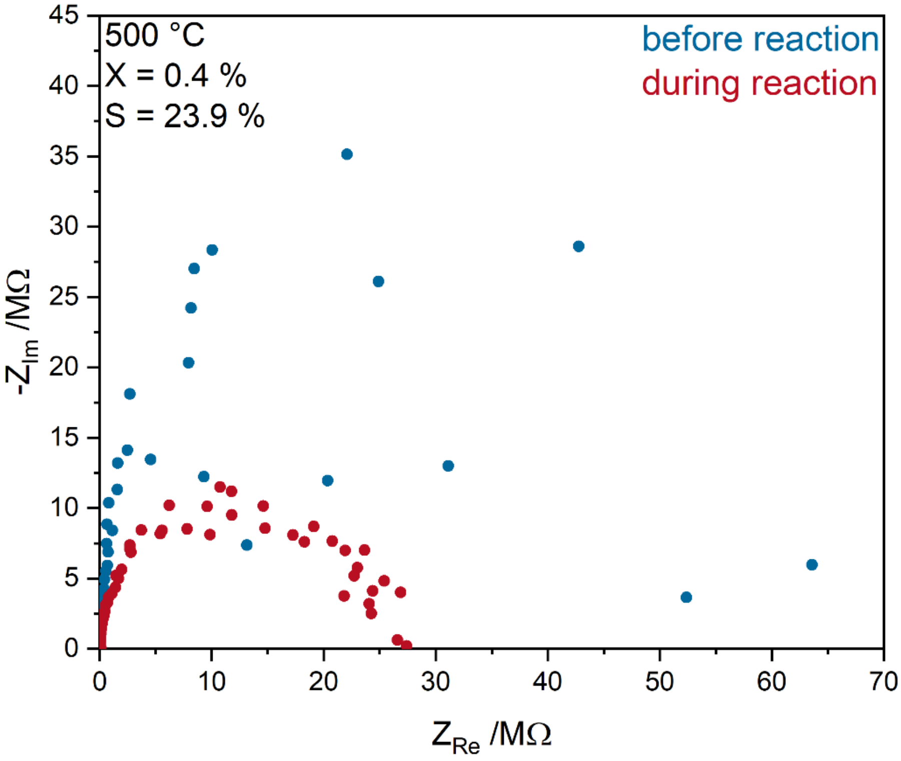

Supplementary Figure 10: Operando impedance spectra of MoO_3 at 500 °C in a feed of 12.5% O_2/12.5% C_3H_8/He, compared to oxidative conditions (12.5% O_2/He).

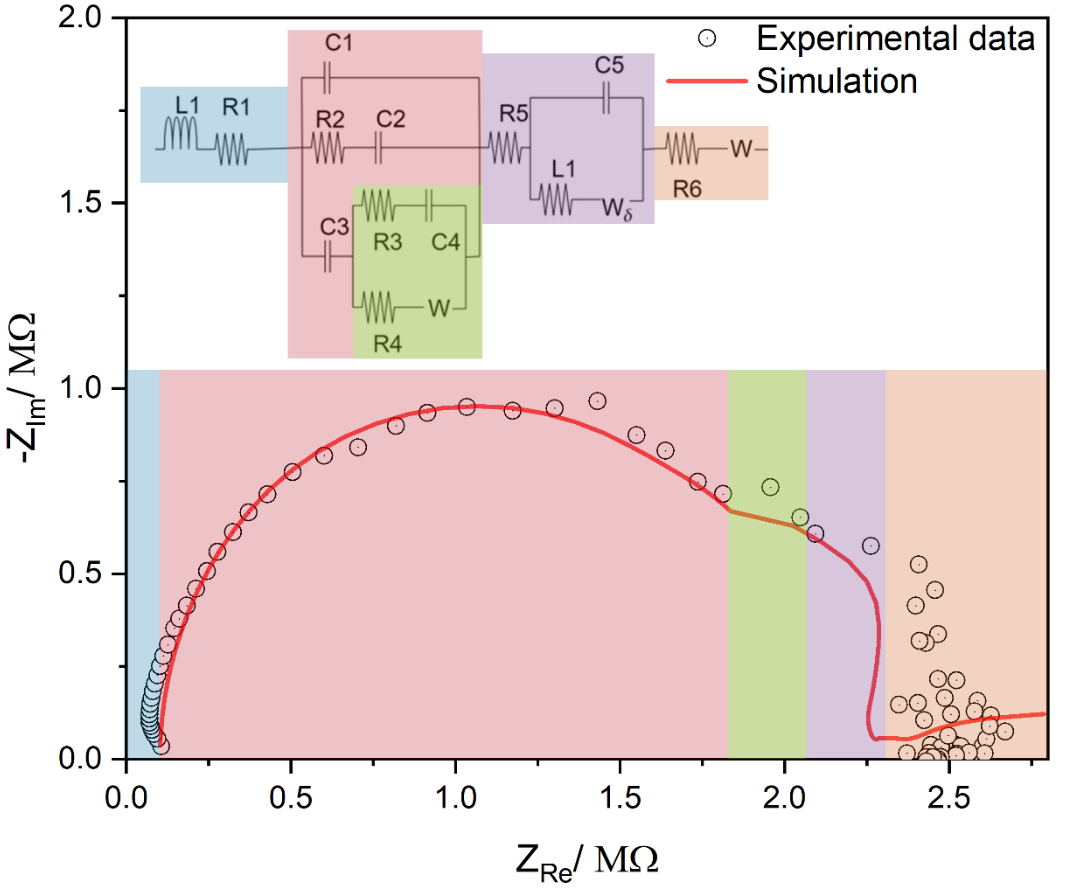

Supplementary Figure 11: Experimental impedance data (points) of $Fe_2(MoO_4)_3$ recorded under reactive conditions (12.5% O_2/12.5% C_3H_8/He) at 500°C together with the results from equivalent circuit fitting (red line). The parts pf the equivalent circuit corresponding to the skin-effect, water creation, protonation and diffusion, as well as separation of $Fe_2(MoO_4)_3$ are indicated by different colors. For details see text.

Supplementary Discussion 2

The impedance spectrum of $Fe_2(MoO_4)_3$ at 500 °C under reactive conditions is composed of multiple elements separated into four sub-circuits. Each one is divided into ohmic, kinetic and ohmic/kinetic contributions. The ohmic parts represent the materials response, while the kinetic parts describe effects due to the occurring reaction.

The sub-circuit on the left (low impedance/high frequency) is composed of an induction (L) and a resistance (R) element in series (blue). When the indirect band gap of 2.7 eV of $Fe_2(MoO_4)_3$ is considered, the material can be described as a semi-conducting oxide,[4] which therefore implies impaired conductivity at room temperature. This causes the alternating current from the AC-potentiostatic impedance experiment to generate charge at the surface, leading to a high accumulation of current, which is described as the skin effect and is often fitted by L-R elements, even though, in some cases, this part is subtracted from the data.[5] This inductive contribution was minimal in our case, yielding a value of 1.39E-03 H after fitting while the electrode resistance was more significant in comparison, yielding a value of R1=101 kΩ, which is expected for a solid-state system with diminished conductivity.[6]

Following the impedance acquisition path from left (high frequency) to right (low frequency) the measurement time increases for each acquired point. The second sub-circuit of the impedance spectrum (red) is composed of four parts, each of which corresponds to different phases present in addition to $Fe_2(MoO_4)_3$ before and/or due to the reaction, that is, MoO_x, $FeMoO_4$ and Fe_2O_3, as determined by separate measurements (see main text and previous studies).[7,8] MoO_x and $FeMoO_4$ have the largest impact on the spectrum of $Fe_2(MoO_4)_3$; note that the contribution of MoO_x during the reaction has already been observed during transient IR measurements. The exact allocation of each phase to their electrical parts in the second sub-circuit is proposed to be a mixture. This corresponds to the C1-R2 parallel circuit in series with C2, and C2 is in parallel with C3. This C-C element describes the mixed character of MoO_x as Mo(V) and Mo(VI) corresponding to the induced oxygen transport initiated by the reaction and hydrogen transfer upon the first C-H breakage or water reduction.[9]

The third sub-circuit (containing R3, R4, C4, and the Warburg element; green) also describes oxygen transport in compliance with the Mars–van Krevelen mechanism and Frenkel equilibrium: $M_M^x + O_O^x \leftrightarrow M_M^x + O_i'' + V_O^{\cdot\cdot}$. Here, the Kröger–Vink

notation describes the presence of oxygen vacancies within the metal oxide to be responsible for the oxygen conduction. As part of the above-mentioned phase separation, the more-conductive $FeMoO_4$ is generated, which causes less charge accumulation. Therefore, the second semi-circle in the impedance spectrum is reduced in size since $FeMoO_4$ is more conductive (even at room temperature) than $Fe_2(MoO_4)_3$ at temperatures between 370 and 900 K.[10,11]

The last semi-circle in the lower-frequency region (purple) corresponds to the kinetic/ohmic contribution. Here, the reaction according to the Mars–van Krevelen mechanism occurs. An interesting electrical component, leading to a significantly improved fit (see Supplementary Figure 7, where only the oxygen transport is regarded), is a proton transport element in the form of R5, which is fully consistent with the transient hydrogen transfer suggested by the ME-DRIFTS data. The impedance data further indicates the diffusion and storage to be mixed according to the analysis performed by Chaparro et al.[12]. This is also the region of mass-transport loss, which indicates the release of compounds from the surface, which were previously diffusing out from the bulk and sub-surface. Here, we propose a mass-transport loss due to water, which is a product of the reaction.[13]

The last part of the spectrum (orange) in the lowest-frequency region corresponds to the mixed mass-transport loss (R) and compensation by the ongoing reaction (Warburg element W). The last R+W contribution (orange) corresponds to a mass-transfer effect, while the previous contribution (purple) describes the kinetic contribution from the reaction itself.

To further support the necessity of the proposed additional effects to oxygen transport, we performed a fit to the same impedance spectrum by taking only oxygen transport into account. The results are shown in Supplementary Figure 8. If only oxygen transport based on RC-circuits is considered, the fit does not converge completely. This behavior is caused by the lack of diffusive effects, hydrogen transport and the inductive elements arising from the skin effect and vacancy displacement according to the Frenkel equilibrium. For the fit shown in Supplementary Figure 8, data points strongly deviating from the spectrum were removed, but the quality of the fit is still poor. This behavior strongly indicates that oxygen transport is not the only phenomenon that needs to be taken into account to describe the impedance data of $Fe_2(MoO_4)_3$.

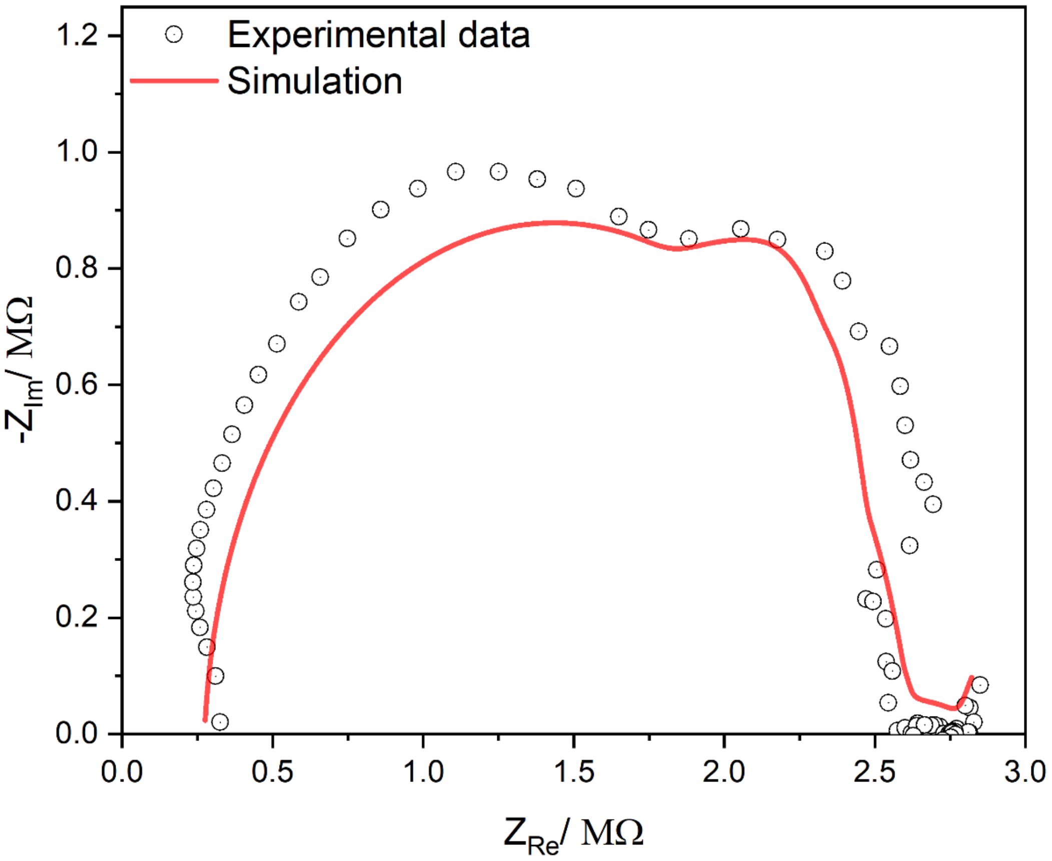

Supplementary Figure 12: Experimental impedance data (points) of $Fe_2(MoO_4)_3$ recorded under reactive conditions (12.5% O_2/12.5% C_3H_8/He) at 500°C together with the results from equivalent circuit fitting (red line) based on oxygen transport effects only. For details see text.

Supplementary References

1. Urakawa, A., Bürgi, T. & Baiker, A. Sensitivity enhancement and dynamic behavior analysis by modulation excitation spectroscopy: Principle and application in heterogeneous catalysis. *Chem. Eng. Sci.* **63,** 4902–4909; 10.1016/j.ces.2007.06.009 (2008).

2. Srinivasan, P. D., Patil, B. S., Zhu, H. & Bravo-Suárez, J. J. Application of modulation excitation-phase sensitive detection-DRIFTS for in situ /operando characterization of heterogeneous catalysts. *React. Chem. Eng.* **4,** 862–883; 10.1039/C9RE00011A (2019).

3. Sejkora, J. *et al.* A Raman spectroscopic study of a hydrated molybdate mineral ferrimolybdite, $Fe_2(MoO_4)_3 \cdot 7\text{-}8H_2O$. *Spectrochim. Acta A Mol. Biomol. Spectrosc.* **130,** 83–89; 10.1016/j.saa.2014.03.112 (2014).

4. Parveen, S. *et al.* Synthesis, characterization and photocatalytic performance of iron molybdate ($Fe_2(MoO_4)_3$) for the degradation of endosulfan pesticide. *Mater. Res. Express* **7,** 35016; 10.1088/2053-1591/ab73fa (2020).

5. Boukamp, B. A., Rolle, A., Vannier, R. N., Sharma, R. K. & Djurado, E. Electrostatic spray deposited $Ca_3Co_4O_{9+\delta}$ and $Ca_3Co_4O_{9+\delta}/Ce_{0.9}Gd_{0.1}O_{1.95}$ cathodes for SOFC. *Electrochim. Acta* **362,** 137142; 10.1016/j.electacta.2020.137142 (2020).

6. Laschuk, N. O., Easton, E. B. & Zenkina, O. V. Reducing the resistance for the use of electrochemical impedance spectroscopy analysis in materials chemistry. *RSC Adv.* **11,** 27925–27936; 10.1039/d1ra03785d (2021).

7. Bowker, M. Rules for Selective Oxidation Exemplified by Methanol Selective Oxidation on Iron Molybdate Catalysts. *Top. Catal.* **58,** 606–612; 10.1007/s11244-015-0399-4 (2015).

8. Oefner, N. *et al.* Activity, Selectivity and Initial Degradation of Iron Molybdate in the Oxidative Dehydrogenation of Ethanol. *ChemCatChem* **14**; 10.1002/cctc.202101219 (2022).

9. Bredar, A. R. C., Chown, A. L., Burton, A. R. & Farnum, B. H. Electrochemical Impedance Spectroscopy of Metal Oxide Electrodes for Energy Applications. *ACS Appl. Energy Mater.* **3,** 66–98; 10.1021/acsaem.9b01965 (2020).

10. Suresh, G., Shakkeel, N. K. & Kalaiselvi, N. $FeMoO_4$ nanorods anchored on graphene sheets as a potential anode for high performance sodium ion batteries. *J. Alloys Compd.* **877,** 160306; 10.1016/j.jallcom.2021.160306 (2021).

11. Forzatti, P., Villa, P. L. & Mari, C. M. Electrical conductivity of polycrystalline $Fe_2(MoO_4)_3$. *Mater. Chem. Phys.* **10,** 385–391; 10.1016/0254-0584(84)90099-3 (1984).

12. Chaparro, A. M., Duque, L. & Folgado, M. A. Analytical Model for the Hydrogen Transport Impedance at a PEMFC Anode. *Meet. Abstr.* **MA2022-02,** 2521; 10.1149/MA2022-02502521mtgabs (2022).

13. Jalani, N. H. *et al.* Performance analysis and impedance spectral signatures of high temperature PBI–phosphoric acid gel membrane fuel cells. *J. Power Sources* **160,** 1096–1103; 10.1016/j.jpowsour.2006.02.094 (2006).
